# Text-Guided Visual Representation Optimization for Sensor-Acquired Video Temporal Grounding

**DOI:** 10.3390/s25154704

**Published:** 2025-07-30

**Authors:** Yun Tian, Xiaobo Guo, Jinsong Wang, Xinyue Liang

**Affiliations:** School of Optoelectronic Engineering, Changchun University of Science and Technology, Changchun 130022, China

**Keywords:** video temporal grounding, cross-modal learning, cross-attention, contrastive learning, representation optimization

## Abstract

Video temporal grounding (VTG) aims to localize a semantically relevant temporal segment within an untrimmed video based on a natural language query. The task continues to face challenges arising from cross-modal semantic misalignment, which is largely attributed to redundant visual content in sensor-acquired video streams, linguistic ambiguity, and discrepancies in modality-specific representations. Most existing approaches rely on intra-modal feature modeling, processing video and text independently throughout the representation learning stage. However, this isolation undermines semantic alignment by neglecting the potential of cross-modal interactions. In practice, a natural language query typically corresponds to spatiotemporal content in video signals collected through camera-based sensing systems, encompassing a particular sequence of frames and its associated salient subregions. We propose a text-guided visual representation optimization framework tailored to enhance semantic interpretation over video signals captured by visual sensors. This framework leverages textual information to focus on spatiotemporal video content, thereby narrowing the cross-modal gap. Built upon the unified cross-modal embedding space provided by CLIP, our model leverages video data from sensing devices to structure representations and introduces two dedicated modules to semantically refine visual representations across spatial and temporal dimensions. First, we design a Spatial Visual Representation Optimization (SVRO) module to learn spatial information within intra-frames. It selects salient patches related to the text, capturing more fine-grained visual details. Second, we introduce a Temporal Visual Representation Optimization (TVRO) module to learn temporal relations from inter-frames. Temporal triplet loss is employed in TVRO to enhance attention on text-relevant frames and capture clip semantics. Additionally, a self-supervised contrastive loss is introduced at the clip–text level to improve inter-clip discrimination by maximizing semantic variance during training. Experiments on Charades-STA, ActivityNet Captions, and TACoS, widely used benchmark datasets, demonstrate that our method outperforms state-of-the-art methods across multiple metrics.

## 1. Introduction

**Figure 1:**
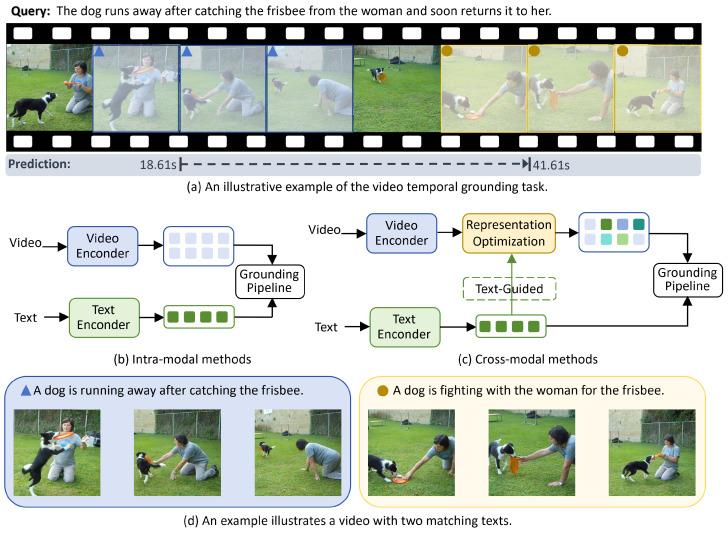
Illustration of the video temporal grounding task and representative modeling strategies: (**a**) a definition example of the VTG task, where the goal is to retrieve a semantically consensual segment from an untrimmed video given a natural language query; (**b**) a representative intra-modal architecture in which video and textual modalities are processed independently, with fusion deferred to the matching stage; (**c**) the proposed text-guided cross-modal modeling framework, which leverages textual semantics to optimize visual representation and concentrate on spatiotemporal video contents that are semantically aligned with the query; (**d**) examples of how the same video responds to two semantically similar but focus-divergent queries.

The rapid proliferation of video data, largely collected through camera-based sensing systems, has elevated video analysis to a central focus in computer vision research. While conventional tasks such as video classification [1,2] and action localization [3,4] have advanced semantic understanding, they depend heavily on predefined label sets and are ill-suited for open-ended querying. Recent advances have introduced natural language as a flexible query modality, enabling users to articulate semantic intent with greater freedom. This conceptual shift has given rise to the research task of video temporal grounding (VTG), which aims to semantically localize segments from untrimmed video signals acquired via visual sensors. As illustrated in Figure 1a, the goal is to identify the temporal segment within an untrimmed video that best aligns with a user-provided textual query [5]. As a canonical task in multimodal learning, VTG has found widespread application in video retrieval, event parsing, and intelligent surveillance. It also serves as a structural foundation for higher-level vision–language tasks such as video dialogue [6], video relationship detection [7,8,9], and video question answering [10,11,12]. By grounding semantic queries in real-world sensor-acquired video data, VTG constitutes a critical bridge between computer vision and natural language processing, promoting intelligent scene perception.

Most existing VTG methods [13,14,15,16,17] construct feature representations independently within either the video or text modality. As illustrated in Figure 1b, these intra-modal approaches employ separate encoders for each modality, extracting unimodal features that are passed to downstream modules for segment generation and boundary prediction. However, because visual features are extracted in isolation from sensor data without semantic conditioning, aligning them effectively with textual queries becomes inherently challenging. The absence of cross-modal interaction prevents an effective representation of semantically discriminative sensor data, limiting the model’s responsiveness to subtle variations in natural scenes. Consequently, models become overly dependent on downstream modules for semantic compensation, weakening early-stage perceptual sensitivity and ultimately undermining the interpretability and precision of cross-modal alignment.

Unlike conventional VTG methods that construct representations independently within each modality, our approach adopts a cross-modal paradigm to more effectively bridge the semantic gap between video and text. Given that textual descriptions often provide more precise semantic cues than raw visual signals captured by sensors [18], we leverage textual guidance to semantically reshape cross-modal visual representations via a text-guided optimization module, as illustrated in Figure 1c. By introducing cross-modal interaction during the visual feature construction stage, our model strengthens the semantic alignment of representations with respect to the query’s intent. This mechanism enables sensor-acquired visual features to undergo early semantic filtering and exhibit stronger task awareness prior to fusion.

Natural language descriptions inherently encode rich temporal structures and fine-grained spatial interactions, making them especially effective for guiding semantic interpretation of sensory visual data. Conventional cross-modal approaches often fall short in capturing such nuanced semantics. Specifically, a natural language query typically refers to a continuous sequence of frames while also conveying intent about inter-frame transitions and the localization of salient regions. As shown in Figure 1d, different textual descriptions of the same sensor video stream can induce distinct patterns of attention. For example, the blue query highlights a specific action unit, “a dog biting the frisbee,” concentrating attention on the moment of contact. In contrast, the brown query emphasizes “dog and woman scrambling for the frisbee,” leading to broader attention over dynamic interactions. Without explicit textual guidance, these distinctions are often lost in low-level encodings of sensor inputs. Therefore, to improve the perceptual accuracy of intelligent sensing systems, visual representation learning must incorporate mechanisms that allow sensor-derived features to adapt dynamically to query-level semantics.

Based on the preceding analysis, we propose a text-guided visual representation optimization framework that incorporates two specialized modules to identify text-relevant video frames and internal patches for constructing discriminative visual representations tailored to video temporal grounding. The Spatial Visual Representation Optimization (SVRO) module identifies spatially salient regions in sensor-acquired video frames that exhibit strong semantic correspondence with the textual query. Unlike prior approaches [19,20,21] that rely solely on visual-domain saliency detection, SVRO conditions patch selection on textual input, computing attention scores between visual patches and query tokens using a cross-attention mechanism. The top-K semantically relevant patches are retained to form the final representation, suppressing irrelevant background and noise. The Temporal Visual Representation Optimization (TVRO) module learns temporal alignment between candidate frames and the query using a contrastive learning strategy. A temporal triplet loss enforces semantic separation between text-relevant and -irrelevant video frames, encouraging the model to emphasize continuous key frames while discarding semantic outliers. These modules work in tandem to model the spatiotemporal semantics embedded in the video signals acquired through sensing systems, producing task-aware features for candidate moment generation and boundary prediction.

We conduct systematic evaluations of the proposed method on three widely used benchmark datasets: Charades-STA [5], ActivityNet-Captions [22], and TACoS [23]. The results demonstrate that our method consistently surpasses existing baselines across multiple standard evaluation metrics. Notably, on the ActivityNet-Captions dataset, Rank@1 at IoU = 0.5 reaches 49.18% and an mIoU of 45.76%. Additional ablation studies validate the effectiveness of the proposed modules and confirm the essential role of text-guided optimization in enhancing the semantic quality of sensor-derived video representations.

The main contributions of this work are summarized as follows:

- We propose a text-guided visual representation optimization framework for video temporal grounding, which introduces cross-modal interaction during the visual encoding stage. This framework reshapes visual representations derived from sensor-acquired video signals under semantic conditioning by natural language queries.
- We design spatial and temporal optimization modules that identify semantically relevant patches and frames from sensed video streams. These modules enhance the discriminability of visual features at both intra-frame and inter-frame levels, contributing to more precise semantic grounding.
- Extensive experiments on three public benchmarks demonstrate that our method consistently outperforms existing approaches. Furthermore, the proposed modules exhibit strong generalization and can be seamlessly integrated into various VTG architectures, offering performance improvements without modifying the core structure. These results highlight the method’s potential in intelligent visual sensing and semantic-level video interpretation.

## 2. Related Work

### 2.1. Video Temporal Grounding

Temporal localization in untrimmed videos primarily encompasses two subfields: temporal action localization and video temporal grounding. The former aims to identify the temporal boundaries of predefined action categories, making it suitable for action detection within a closed category space, yet it faces considerable challenges when handling open-ended natural language instructions [3,24]. To address this constraint, Gao et al. [5] and Hendricks et al. [25] first proposed the video temporal grounding task in 2017, with the goal of precisely locating temporal boundaries in video that align with a natural language query. Most existing VTG methods [13,14,15,16,17] follow an intra-modal modeling paradigm, where visual and textual features are independently encoded during the representation stage and subsequently passed to downstream modules for candidate moment modeling and boundary prediction. While this strategy allows for coarse-grained semantic alignment via temporal modeling or matching functions, the absence of cross-modal interaction makes it difficult for visual representations to respond dynamically to language queries and ultimately restricts the model’s capacity for fine-grained detail modeling and precise grounding. These untrimmed video signals are typically captured through camera-based sensing systems, making VTG a critical task for interpreting and structuring sensor-acquired visual data using high-level textual supervision.

In light of these limitations, recent studies have increasingly turned to cross-modal modeling paradigms that incorporate language-guided supervision and symmetric interaction mechanisms to enhance the semantic fidelity of visual representations. One line of research focuses on language-guided visual representation learning, such as LGI [26], PS-VTG [27], and VDI [28], which segment natural language queries into semantic phrases or sub-queries to guide the visual encoding of candidate clips, thereby achieving fine-grained alignment between textual cues and spatial regions. Another line of work explores bidirectional regulation mechanisms, enabling visual features to modulate the language encoding process in reverse. Representative methods such as CBLN [29] and SeqPAN [30] employ symmetric attention and gated control strategies to refine the semantic mapping process. While these methods enhance the semantic consistency of visual representations, two critical limitations persist. First, their modeling granularity is typically restricted to the frame level, lacking intra-frame spatial saliency modeling. This omission hinders the identification of text-relevant regions within individual frames and results in blurred semantic distinctions. Second, they fail to effectively filter redundant frames, allowing irrelevant content to persist in the visual representation. This inclusion leads to semantic misalignment and reduces the discriminative capacity of the model. In contrast, our work extends the modeling mechanism along two key dimensions. In the spatial dimension, we introduce a salient patch selection strategy that leverages a text-guided mechanism to identify semantically relevant intra-frame regions and achieve fine-grained spatial alignment. In the temporal dimension, we build a redundancy suppression path to suppress the influence of semantically irrelevant frames and enhance the temporal discriminability of visual representations, thereby improving overall focus on the textual semantics.

### 2.2. Cross-Modal Learning for VTG

In real-world scenarios, actions and events are often conveyed through multiple modalities, with vision–language correspondence playing a central role in semantic modeling. As natural language increasingly emerges as the dominant medium of human–computer interaction, the collaborative modeling of visual and textual modalities has become a cornerstone in cross-modal learning research [31,32,33]. In sensor-acquired video data, semantic structures, such as object interactions and temporal scene transitions, are often entangled within raw pixel streams. The ability to structure such information via text-guided modeling significantly enhances semantic-level sensing and scene understanding. In recent years, the widespread adoption of Transformer architectures has significantly advanced language understanding and representation learning, and their core attention mechanisms have been progressively integrated into multimodal tasks. These mechanisms have shown strong efficacy in fine-grained problems such as video temporal grounding. The majority of existing studies adopt the cross-attention mechanism [34] as the foundational framework for vision–language fusion, leveraging the modeling of inter-modal dependencies to enhance semantic coherence and alignment precision. Representative methods such as VSLNet [35], GDP [36], CBLN [29], SeqPAN [30], and MRTNet [16] utilize cross-attention strategies during modality fusion, effectively enabling semantic alignment between visual and textual features.

While many existing methods incorporate the cross-attention mechanism after completing visual feature encoding, this “post hoc interaction” modeling paradigm improves matching to some extent but lacks textual semantic guidance in the early stages of visual encoding, which results in insufficient responsiveness of visual features to key semantic details. This makes it difficult to structurally model spatial saliency and suppress temporal redundancy, thereby limiting overall cross-modal expressiveness. To address the aforementioned modeling limitations, we propose advancing the cross-attention mechanism into the visual encoding stage and coupling it with text to form a unified text-guided aggregator. With respect to spatiotemporal video content, the text-guided aggregator fuses semantically salient intra-frame patches with textual context to construct informed visual representations, suppressing irrelevant frame sequences and preventing redundant information from contaminating the visual feature construction. This early-stage guidance is particularly valuable when interpreting complex dynamic scenes recorded by visual sensors in real-world environments. Compared with conventional approaches that focus on post-fusion semantic alignment, our method introduces early-stage structural alignment and embeds semantic constraints during initial encoding, concentrating structural changes in the low-level representation generation process, fundamentally enhancing the structural responsiveness of visual features to textual semantics, and providing a unified modeling solution for spatial saliency encoding and temporal noise suppression.

### 2.3. Contrastive Learning for VTG

Contrastive learning, as a representative self-supervised learning paradigm, aims to reduce the distance between semantically aligned samples while increasing the distance between misaligned ones. This approach has demonstrated strong effectiveness in representation learning and has been widely adopted in cross-modal retrieval [37,38] and spatiotemporal modeling tasks [39,40]. Among the most influential contrastive learning frameworks, CLIP [41] constructs a joint visual–textual embedding space by training on large-scale image–caption pairs, exhibiting strong capabilities in cross-modal alignment and generalization. CLIP4Clip [42] extends CLIP to video–language tasks by generating frame-level visual representations conditioned on textual semantics. CLIP-ViP [43] further introduces a staged training strategy to address semantic shifts between video captions and natural language queries. These studies collectively suggest that incorporating contrastive learning into video–text joint modeling can significantly enhance cross-modal consistency and improve downstream discrimination performance. This paradigm is especially effective when learning from video signals captured by camera-based sensing systems, where supervised labels are often sparse or absent, enabling semantic representations to emerge directly from raw sensory input.

In video temporal grounding, contrastive learning is widely employed to enhance the discriminative power of cross-modal alignment and to sharpen semantic focus. For example, MMN constructs a 2D candidate moment feature map and introduces a contrastive loss to strengthen text-to-candidate matching, thereby improving the model’s response to semantically salient segments [13]. D3G proposes a semantic-aligned grouped contrastive learning mechanism (SA-GCL), which partitions intra-video and inter-video sample groups and utilizes a Gaussian adjustment module for dynamic modeling of the visual–semantic distribution, building hierarchical contrastive paths across videos to enhance segment-level discrimination [17]. These methods validate the feasibility and effectiveness of contrastive learning in VTG from different perspectives. Building upon the contrastive capabilities of CLIP, our method further embeds contrastive learning into the Temporal Visual Representation Optimization (TVRO) module. This module constructs a contrastive structure based on the semantic relation between candidate frames and text, treating semantically relevant frames as positive samples and irrelevant ones as negative samples. A temporal triplet loss is introduced to enlarge the semantic response gap between the two frame groups, guiding the model to focus on key frame sequences while suppressing redundant frames. In addition, we design a clip–text self-supervised contrastive loss, aimed at capturing semantic distinctions among candidate segments throughout training. By leveraging contrastive supervision over sensor-acquired video signals, our model constructs semantically aligned representations with minimal reliance on annotated data, enabling efficient learning from raw visual inputs.

## 3. Methodology

**Figure 2:**
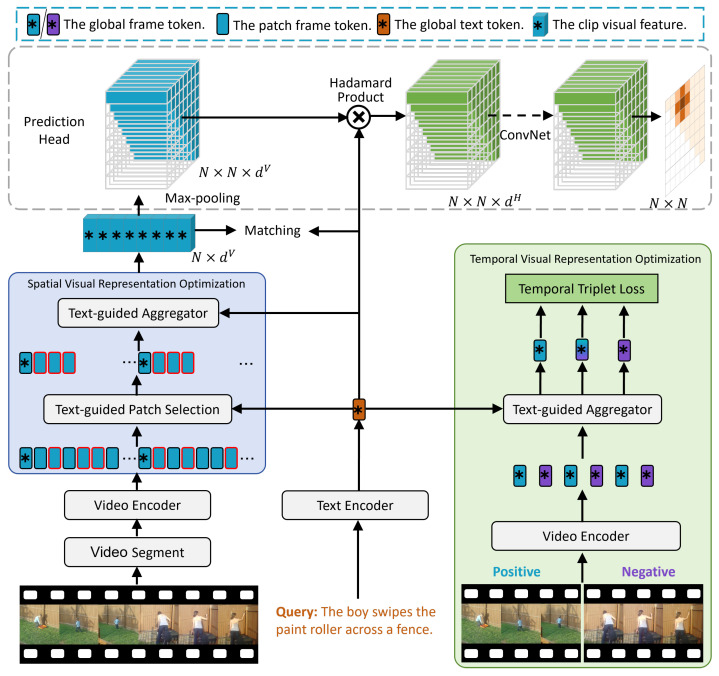
Overview of the proposed text-guided visual representation optimization framework for video temporal grounding. The proposed framework consists of two core components: Spatial Visual Representation Optimization (SVRO) and Temporal Visual Representation Optimization (TVRO). Given an untrimmed video and a language query, we first extract video representations via CLIP encoders. SVRO selects text-relevant patch tokens within each frame and aggregates them using text-aggregator. TVRO identifies text-relevant key frames and employs a temporal triplet loss to refine the temporal focus of the model. The optimized visual representations are subsequently fed into the prediction head for candidate moment construction and localization.

This section systematically introduces the proposed text-guided visual representation optimization method. As illustrated in Figure 2, the architecture consists of three main stages: First, the CLIP encoder is used to embed both video and text inputs into a shared semantic space, generating a unified visual–textual representation. Second, the visual representation is optimized in both spatial and temporal dimensions under the guidance of textual semantics. Finally, based on the optimized visual representations, candidate moments are generated, and temporal boundaries are localized.

### 3.1. Problem Definition

Given an untrimmed video *V* and a natural language query *S*, the objective is to retrieve a temporal segment *M* from *V* that is semantically aligned with *S*. Formally, the query sentence is denoted as $S={\left\{s_{i}\right\}}_{i=0}^{l^{s}-1}$, where $s_{i}$ denotes the i-th word, and $l^{S}$ is the total number of words in the query. The video *V* is represented as a sequence of frames, denoted as $V={\left\{x_{i}\right\}}_{i=0}^{l^{V}-1}$, where $x_{i}$ is the i-th video frame, and $l^{V}$ denotes the total number of frames. The target temporal segment is denoted as $M=[x_{i},x_{j}]$ with $0\leq i\leq j<l^{V}$, and it is required to be semantically consistent with the given query *S*.

### 3.2. Preparation

Given an input video stream, we first segment it into *N* consecutive video clips, denoted as $V={\left\{c_{i}\right\}}_{i=0}^{N-1}$, where each clip $c_{i}$ consists of *T* consecutive frames, formally defined as $c_{i}={\left\{x_{k}\right\}}_{k=nT}^{(n+1)T-1}$. These clips serve as the fundamental units for candidate moment construction. To construct a unified multimodal representation space, CLIP [41] is adopted as the backbone to encode both video and text modalities. For the textual modality, the given natural language query *S* is encoded using the CLIP text encoder, producing a token sequence $\left\{t_{\left[\mathrm{SOS}\right]},\;t_{1},\;t_{2},\;\ldots ,\;t_{l^{S}},\;t_{\left[\mathrm{EOS}\right]}\right\}$, where $t_{\left[SOS\right]}$ and $t_{\left[EOS\right]}$ denote the start and end tokens, respectively. We use $t_{\left[\mathrm{EOS}\right]}\in R^{d^{S}}$ as the global text representation of the sentence, denoted as *t*. For each segmented video clip $c_{i}$, we uniformly sample *m* frames and divide each frame into non-overlapping patches. These patches are then fed into the CLIP visual encoder to extract local visual features. The [CLS] tokens $v_{\left[CLS\right]}$ generated from each frame are aggregated into the set $\left\{v_{1},v_{2},\ldots ,v_{m}\right\}$. Here, $v_{i}\in R^{d^{V}}$ denotes the global visual semantic vector of the i-th frame. This set forms the initial visual representation of the video clip for subsequent cross-modal alignment.

However, the initial visual representations encoded by CLIP often contain redundant regions that are semantically unrelated to the query, which may hinder accurate alignment if directly used in cross-modal fusion. Inspired by [18], we adopt a text-guided mechanism to enhance the semantic relevance of visual representations. We design a text-guided aggregator to perform cross-modal-guided aggregation over frame-level visual representations, which combines cross-attention with a feed-forward network. Specifically, given a paired text and video clip, with the text representation t serving as the query and the visual features treated as the keys and values, cross-attention is applied as shown in Equation (1) to capture the most text-relevant visual regions:

(1) $$\mathrm{CrossAttn}\left(k,q\right)=\mathrm{softmax}\left(\frac{\left(qW_{q}\right){\left(kW_{k}\right)}^{⊤}}{\sqrt{d_{k}}}\right)\left(kW_{v}\right)$$

where $W_{q}$ , $W_{k}$, and $W_{v}$ are the projection matrices in cross-attention. The attention-weighted features are then passed through a feed-forward network to further enrich their representational capacity, producing the final visual representation $v_{t}\in R^{d_{V}}$ :

(2) $$v_{t}=\mathrm{TextAggr}\left(\left\{v_{1},v_{2},\ldots ,v_{m}\right\},\;t\right)=\mathrm{FFN}\left(\mathrm{CrossAttn}(\left\{v_{1},v_{2},\ldots ,v_{m}\right\},\;t)\right)$$

where TextAggr represents the text-guided aggregator. FFN(·) denotes a feed-forward neural network. In contrast to conventional visual encoders that extract static representations, the text-guided aggregator facilitates text-guided selection and the reweighting of visual patches, thereby directing the visual representation toward regions that exhibit high semantic relevance to the query. By retaining essential visual content and suppressing irrelevant regions, the model achieves more precise semantic alignment in the early encoding stage.

### 3.3. Spatial Visual Representation Optimization

**Figure 3:**
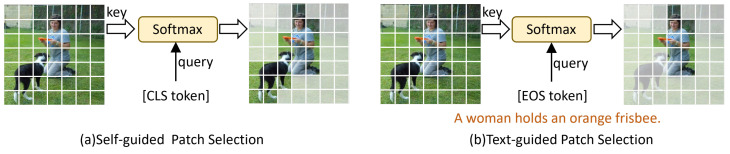
Comparison between self-guided and text-guided patch selection mechanisms. (**a**) Self-guided patch selection uses the [CLS] token to identify salient patches within a frame. While it highlights both the woman and the dog, the dog is irrelevant to the query and thus constitutes redundant information. (**b**) Text-guided patch selection uses text tokens to identify patches related to the query. When the query specifically targets the woman, the selection excludes the dog and focuses on patches relevant to the query.

In previous work [44,45,46], the global features of each frame are typically used to construct visual representations, which hinders the model’s capacity to capture fine-grained semantic cues within localized spatial regions. In practice, each frame output by the visual encoder contains multiple patch tokens, many of which are either underused or entirely disregarded. These patch tokens, however, encapsulate rich local visual information and offer a more detailed depiction of the scene. Given the large number of patches per frame, most of which are semantically redundant, selectively identifying informative patches becomes essential for enhancing spatial representations. A straightforward approach is to use the global [CLS] token to select the most informative patch tokens within each frame. As shown in Figure 3a, patches with high similarity to the [CLS] token are considered important, which results in retaining them as visual representations within the frame. Considering that natural language queries typically focus on specific patches of a frame, selecting patches that are semantically relevant to the text is crucial for improving cross-modal alignment. To this end, we propose a text-guided patch selection mechanism, as illustrated in Figure 3b. It effectively enhances the semantic responsiveness and spatial discrimination of visual features while mitigating the impact of redundant content.

Specifically, for the i-th frame within a given video clip, the corresponding patch feature set is denoted as ${\left\{p_{i,j}\right\}}_{j=1}^{k}$, where $p_{i,j}\in R^{d_{V}}$ denotes the *j*th patch of the *i*-th frame, and *k* is the number of patches per frame. To evaluate the semantic relevance between each patch and the textual query, we adopt a cross-modal attention mechanism to compute attention scores and then normalize and rank them. The top *K* patches most relevant to the query are selected, and they are defined as

(3) $${\left\{p_{i,j}^{*}\right\}}_{j=1}^{k}={\mathrm{TopK}}_{j}\left(\mathrm{softmax}\left(\frac{\left(tW_{q}\right){\left(p_{i,j}W_{k}\right)}^{⊤}}{\sqrt{d_{k}}}\right)\right)$$

where ${\left\{p_{i,j}^{*}\right\}}_{j=1}^{K}$ denote the top−K patch tokens from the *i*th frame that are the most semantically aligned with the global textual feature *t*. This mechanism effectively filters out the background patches that are semantically irrelevant to the query while retaining only the most informative and query-relevant patches. However, although solely relying on local patch features improves semantic focus, it may compromise the global structural integrity within each frame, resulting in fragmented visual representations and weakened contextual awareness. To this end, we concatenate the global [CLS] token $v_{i}$ of each frame with its top−K selected patch features to form a fused frame visual representation ${\hat{v}}_{i}$, denoted as

(4) $${\hat{v}}_{i}=\left[v_{i}\;∥\;P_{i}^{*}\right],\;P_{i}^{*}={\left\{p_{i,j}^{*}\right\}}_{j=1}^{k}$$

wherethe [CLS] token $v_{i}$ serves as a global semantic summary, which complements the local patches with missing contextual structural information, achieving a unified modeling of both global and local semantics. Based on this, to further enhance the responsiveness of visual features to textual semantics, we input the concatenated frame visual feature sequence $\left\{{\hat{v}}_{1},{\hat{v}}_{2},\ldots ,{\hat{v}}_{m}\right\}$ into the text-guided aggregator and perform cross-modal interaction with the text representation t to generate a spatial-enhanced visual representation $v^{*}$:

(5) $$v^{*}=\mathrm{TextAggr}\left(\left\{{\hat{v}}_{1},{\hat{v}}_{2},\ldots ,{\hat{v}}_{m}\right\},\;t\right)$$

The optimized visual representation is not only more sensitive to query semantics at the local level but also maintains contextual consistency at the global level. In Section 4.5.2, we further conduct experiments to demonstrate the effectiveness of incorporating text-related patches.

### 3.4. Temporal Visual Representation Optimization

**Figure 4:**
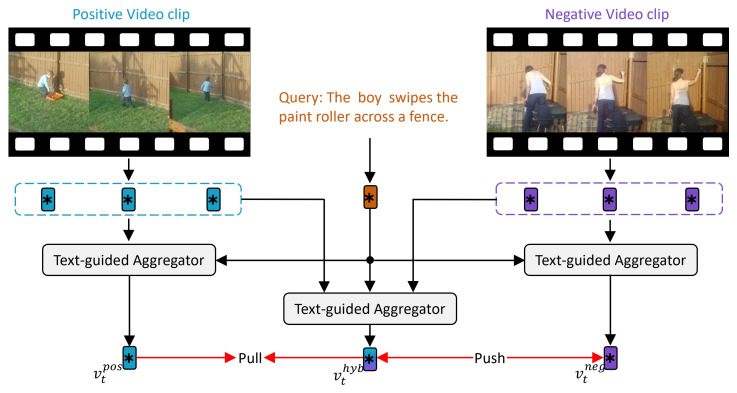
An example of the temporal triplet loss $L_{\mathrm{Tri}}$ employed during training. The text serves as semantic guidance and forms a positive pair with the semantically relevant video clip while forming a negative pair with the semantically irrelevant clip. The text-guided aggregator is used to fuse clip visual features with text semantics, generating the positive feature $v_{t}^{pos}$, the negative feature $v_{t}^{neg}$, and a hybrid representation $v_{t}^{hyb}$ derived from a semantically mixed frame sequence. The temporal triplet loss $L_{\mathrm{Tri}}$ minimizes the distance between $v_{t}^{hyb}$ and $v_{t}^{pos}$ while maximizing the distance between $v_{t}^{hyb}$ and $v_{t}^{neg}$, thereby encouraging the model to focus on frames that are highly aligned with the query semantics and suppress responses to redundant or distracting frames.

Although the text-guided patch selection in the spatial dimension effectively reduces redundant patches, the visual representations still include numerous frames irrelevant to the query in the temporal dimension, such as static backgrounds, action transition frames, or irrelevant event segments. These redundant frames, when incorporated into temporal modeling, compromise the semantic purity of the resulting visual representations, thereby impairing the cross-modality alignment accuracy. To address this, the construction of video clip features should emphasize frames that are semantically consistent with the query. Specifically, query-relevant frames are regarded as positive samples, while the remaining ones are treated as negatives. The final visual representation is then aggregated from the positive frames only. As shown in Figure 4, the proposed TVRO module enhances attention toward positive frames while suppressing the influence of negatives, guiding the visual representation to concentrate on temporally salient segments and improving temporal semantic alignment.

The precise identification of key frames typically requires extensive manual annotation, which is impractical in practical applications. To address this issue, we propose a frame selection strategy based on Intersection over Union (IoU) consistency, enabling the approximate identification of key frames without additional labeling. First, we map the clip index to a temporal interval, as shown in Equation (6):

(6) $$\mathrm{Time}\left(c_{i}\right)=\left[i\cdot \tau ,\;\left(i+1\right)\cdot \tau \right],\;\tau =\frac{L}{N}$$

where *L* is the total duration of the video, and *N* is the number of equally divided video clips. τ is a short duration of each clip determined by the video length and sampling rate. We construct positive and negative clip sets based on their IoU overlap with the ground-truth interval $\left[t_{s},\;t_{e}\right]$. The positive clip set $C^{+}$ and negative clip set $C^{-}$ are defined as follows:

(7) $$\begin{array}{cc} C^{+} & =\{c_{i}|\mathrm{IoU}\left(\mathrm{Time}\left(c_{i}\right),\left[t_{s},t_{e}\right]\right)\geq \theta \},\\ C^{-} & =\{c_{i}|\mathrm{IoU}\left(\mathrm{Time}\left(c_{i}\right),\left[t_{s},t_{e}\right]\right)<\theta \} \end{array}$$

where θ is an empirically determined threshold to ensure a distinct semantic gap between positive and negative samples. Subsequently, we uniformly sample *f* frames from the positive and negative clip sets, constructing a positive frame sequence $\left\{v_{1}^{\mathrm{pos}},\;v_{2}^{\mathrm{pos}},\;\ldots ,\;v_{m}^{\mathrm{pos}}\right\}$ and a negative frame sequence $\left\{v_{1}^{\mathrm{neg}},\;v_{2}^{\mathrm{neg}},\;\ldots ,\;v_{m}^{\mathrm{neg}}\right\}$. We apply the text-guided aggregator to both the positive and negative frame sequences conditioned on the global text feature *t*, resulting in the following cross-modal visual representations:

(8) $$\begin{array}{cc} v_{t}^{\mathrm{pos}} & =\mathrm{TextAggr}(\left\{v_{1}^{\mathrm{pos}},\;v_{2}^{\mathrm{pos}},\;\ldots ,\;v_{f}^{\mathrm{pos}}\right\},\;t),\\ v_{t}^{\mathrm{neg}} & =\mathrm{TextAggr}(\left\{v_{1}^{\mathrm{neg}},\;v_{2}^{\mathrm{neg}},\;\ldots ,\;v_{f}^{\mathrm{neg}}\right\},\;t) \end{array}$$

where $v_{t}^{pos}$ and $v_{t}^{neg}$ represent the cross-modal features of positive and negative frame sequences. We then concatenate the positive and negative frame sequences to form a hybrid sequence $\left\{v_{1}^{\mathrm{pos}},\;\ldots ,\;v_{f}^{\mathrm{pos}},\;v_{1}^{\mathrm{neg}},\;\ldots ,\;v_{f}^{\mathrm{neg}}\right\}$. The text-guided aggregator is further applied to this hybrid sequence to generate the fusion features as follows:

(9) $$v_{t}^{\mathrm{hyb}}=\mathrm{TextAggr}\left(\left\{v_{1}^{\mathrm{pos}},\;\ldots ,\;v_{f}^{\mathrm{pos}},\;v_{1}^{\mathrm{neg}},\;\ldots ,\;v_{f}^{\mathrm{neg}}\right\},\;t\right)$$

where $v_{t}^{hyb}$ should be predominantly generated by positive frames, minimizing the influence of negative frames. To achieve this, we introduce temporal triplet loss as follows:

(10) $$L_{\mathrm{Tri}}=-\log\left(\frac{\exp\left(∥v_{t}^{\mathrm{hyb}}-v_{t}^{\mathrm{neg}}∥\right)}{\exp\left(∥v_{t}^{\mathrm{hyb}}-v_{t}^{\mathrm{pos}}∥\right)+\exp\left(∥v_{t}^{\mathrm{hyb}}-v_{t}^{\mathrm{neg}}∥\right)}\right)$$

where $∥v_{t}^{\mathrm{hyb}}-v_{t}^{\mathrm{neg}}∥$ denotes the L2-norm distance between $v_{t}^{hyb}$ and $v_{t}^{neg}$. This loss treats $v_{t}^{hyb}$ as the anchor, minimizing its distance to the positive frames while maximizing the distance to the negative one. This guides the model to focus on key frames that align with the query semantics. The inter-frame visualization presented in Section 4.5.1 demonstrates the effectiveness of TVRO.

### 3.5. Grounding Head

We follow [47] and construct a prediction head that predicts the best candidate moment based on the optimized visual representations. First, we build up a feature map of candidate moments using the video clip representations ${\left\{v_{i}^{*}\right\}}_{i=0}^{N-1}$. For each candidate moment starting from clip $c_{a}$ to $c_{b}$, we max-pool the corresponding clip features across a specific time span and obtain its feature:

(11) $$v_{\left(a,b\right)}^{M}=\mathrm{maxpool}\left(v_{a}^{*},\;v_{a+1}^{*},\;\ldots ,\;v_{b}^{*}\right),\;0\leq a\leq b\leq N-1$$

We restructure all candidate moments to a 2D temporal feature map, denoted as $V^{M}\in R^{N\times N\times d^{V}}$, where the first two dimensions *N* represent the start and end clip indexes, respectively, while the third one $d^{V}$ indicates the feature dimension. Then, both $V^{M}$ and text feature *t* are projected into a unified subspace by fully connected layers, and they are fused through Hadamard product and $\ell_{2}$ normalization to obtain a cross-modal 2D feature map *F*:

(12) $$F={∥\left(W^{S}\cdot t\cdot 1^{T}\right)⊙\left(W^{M}\cdot V^{M}\right)∥}_{F}$$

where $W^{S}$ and $W^{M}$ are learnable projection matrices, $1^{T}$ is the transpose of an all-ones vector, ⊙ is the Hadamard product, and ${∥\cdot ∥}_{F}$ denotes Frobenius normalization. On the fused 2D feature map *F*, we stack *L* layers of 2D convolution with *H* kernels per layer to gradually perceive more context of adjacent candidate moments while learning the difference between candidate moments. The feature updates for each convolutional layer follow the form

(13) $$F^{\left(l+1\right)}=\mathrm{ReLU}\left(\mathrm{Conv}2D^{\left(l\right)}\left(F^{\left(l\right)}\right)\right),\;l=0,1,\ldots ,L-1$$

Finally, the output 2D temporal map goes through a fully connected layer and a sigmoid function to generate a 2D score map. According to the candidate indicator, all the valid scores on the map are then collected, denoted as $P={\left\{p_{i}\right\}}_{i=1}^{C}$, where *C* is the total number of moment candidates. Each value $p_{i}$ on the map represents the matching score between a moment candidate with the queried sentence. The moment with the highest score is selected as the final grounding result.

### 3.6. Loss Function

We construct a multi-branch joint loss function designed to improve localization accuracy, cross-modal alignment, and key frame semantic selectivity. The overall loss comprises three components: binary cross-entropy loss, cross-modal matching loss, and temporal triplet loss (see Section 3.4).

#### 3.6.1. Binary Cross-Entropy

Following the approach in [47], we adopt the scaled IoU score of each candidate moment as the supervision signal. More precisely, for each candidate moment, we first compute its temporal overlap with the ground-truth interval, denoted as $o_{i}$. The overlap score is then linearly scaled into a soft supervision label $y_{i}$ according to predefined lower and upper thresholds $t_{min}$ and $t_{max}$ as follows:

(14) $$y_{i}=\left\{\begin{array}{cc} 0, & o_{i}\leq t_{\min}\\ \frac{o_{i}-t_{\min}}{t_{\max}-t_{\min}}, & t_{\min}<o_{i}<t_{\max}\\ 1, & o_{i}\geq t_{\max} \end{array}\right.$$

In this way, the label reflects the proximity between the candidate moment and the ground-truth interval. During training, a standard binary cross-entropy loss is used for optimization, defined as

(15) $$L_{\mathrm{BCE}}=\frac{1}{C}\sum_{i=1}^{C}y_{i}\log\left(p_{i}\right)+\left(1-y_{i}\right)\log\left(1-p_{i}\right)$$

where $p_{i}$ is the output score of a moment, and C is the total number of valid candidates.

#### 3.6.2. Cross-Modal Matching

To further enhance semantic discrimination across candidate clips, we introduce a self-supervised contrastive learning strategy centered on clip–text alignment. This approach guides the model to highlight semantically relevant clips while suppressing irrelevant ones, thereby improving the discriminative quality and robustness of temporal localization. Concretely, given the visual representation $v_{i}^{*}$ of each clip and the global query representation *t*, we compute their cosine similarity to quantify the degree of the cross-modal matching score:

(16) $$\mathrm{Sim}\left(c_{i}\right)=\frac{v_{i}^{*}\cdot t}{∥v_{i}^{*}∥\cdot ∥t∥}$$

To obtain a probability distribution over all candidate clips, the similarity scores are normalized using a softmax function:

(17) $$P\left(c_{i}\right)=\frac{exp(Sim\left(c_{i}\right)/\sigma )}{\sum_{j=1}^{N}exp\left(Sim\left(c_{j}\right)/\sigma \right)}$$

where σ is a temperature parameter that controls the sharpness of the distribution. Lower values emphasize fine-grained differences among segments, whereas higher values produce a more uniform distribution. Following previous work, we set σ to 0.07 in all experiments. Based on this distribution, we define the cross-modal matching loss as

(18) $$L_{\mathrm{Match}}=-\frac{1}{N}\sum_{i=0}^{N-1}\log P\left(c_{i}\right)$$

This loss encourages the model to attend to clips that are most relevant to the query and suppress those that are irrelevant. The overall loss function consolidates three complementary optimization goals: moment localization, clip–text alignment, and key frame semantic sensitivity. It is defined as

(19) $$L_{\mathrm{all}}=L_{\mathrm{BCE}}+\alpha \cdot L_{\mathrm{Match}}+\beta \cdot L_{\mathrm{Tri}}$$

The hyperparameters α and β regulate the relative contributions of the cross-modal matching loss and the temporal triplet loss within the unified training objective.

## 4. Experiments

### 4.1. Datasets

To evaluate the performance of our proposed method, we conduct experiments on three challenging video temporal grounding datasets. Notably, all datasets consist of real-world videos collected via camera-based sensing systems, reflecting the typical input form of vision-based sensors in practical scenarios. These video signals serve as representative sensing data sources and are subsequently used for semantic grounding under textual guidance.

Charades-STA [5] is an extended version of the Charades [48] action recognition dataset, tailored explicitly for the task of video temporal grounding. It comprises 5338 videos and 12,408 query–moment pairs in the training set and 1334 videos with 3720 query–moment pairs in the test set. The original Charades dataset was recorded using RGB cameras in indoor environments with participants performing scripted activities. A salient characteristic of this dataset lies in its high semantic density, with each video associated with multiple overlapping or conflicting queries. This results in ambiguous event boundaries and presents a formidable challenge for precise cross-event alignment. On this dataset, we focus on evaluating the discriminative capabilities of the spatial (SVRO) and temporal (TVRO) optimization modules in handling semantically overlapping scenarios.

ActivityNet-Captions [22] is built upon the large-scale video recognition dataset ActivityNet v1.3 [49], originally designed for video captioning tasks and now widely adopted in temporal grounding research. The videos were collected from the internet and were captured using consumer-grade cameras or mobile devices under unconstrained conditions, making the dataset representative of open-world sensor-acquired video content. There are 37,417, 17,505, and 17,031 moment–sentence pairs for training, validation, and testing, respectively. Following the setting of 2D-TAN [47], we report the evaluation result on val2 set. Characterized by broad coverage, extended event durations, and ambiguous temporal boundaries, the dataset provides a rigorous benchmark for assessing a model’s ability to identify semantically salient segments amid complex event distributions. It offers a particularly suitable testbed for evaluating the robustness of our text-guided patch selection strategy under diverse contextual conditions.

TACoS [23] is constructed from videos of human activities in kitchen environments, comprising 127 long videos and 18,818 video–language pairs, annotated with high-quality labels from [50]. The videos were originally recorded in a controlled kitchen setting using fixed-angle RGB cameras, capturing continuous cooking activities from a first-person view. A standard split [5] consists of 10,146, 4589, and 4083 moment–sentence pairs for training, validation, and testing, respectively. Despite its domain specificity, the dataset is characterized by a high semantic density, continuous yet low-variance activity sequences, and substantial visual redundancy with minimal inter-frame semantic variation. This benchmark is primarily utilized to examine the effectiveness of our TVRO module in scenarios with high visual redundancy and low temporal dynamics. Special emphasis is placed on evaluating its capacity for key frame awareness and redundancy suppression through the proposed temporal triplet supervision strategy.

### 4.2. Experimental Settings

#### 4.2.1. Evaluation Metrics

Following existing video grounding works, we evaluate the performance on two main metrics:

mIoU: “mIoU" is the average predicted Intersection over Union over all testing samples. The mIoU metric is particularly challenging for short video moments.

Recall: We adopt “R@n,IoU=m” as the evaluation metric, following [5]. “R@n,IoU=m” represents the percentage of language queries having at least one result whose IoU between the top−n predictions and the ground-truth is larger than *m*. In our experiments, we report the results of n=1 and m∈0.3,0.5,0.7.

The mIoU metric emphasizes the overall quality of temporal alignment, reflecting the boundary precision gains achieved through the spatial and temporal optimization of visual representations. The use of IoU thresholds at 0.3, 0.5, and 0.7 captures varying levels of semantic tolerance, where lower thresholds evaluate general relevance, and higher ones assess fine-grained localization. This design effectively measures how well our method captures both the coarse and precise temporal extents implied by the query.

#### 4.2.2. Implementation Details

To enable a fair comparison with existing baselines in terms of both architecture and training pipeline, we follow the 2D convolutional configuration adopted in [47] for candidate moment modeling and temporal boundary prediction. Specifically, the number of sampled clips *N* is set to 16 for Charades-STA, 64 for ActivityNet Captions, and 128 for TACoS. The number of frames in a clip *T* is set to 4 for Charades-STA and 16 for ActivityNet Captions and TACoS. For the convolutional structure, Charades-STA and TACoS employ an eight-layer network, with a kernel size of 5, whereas ActivityNet Captions adopts a four-layer network, with a kernel size of 9. The dimensionalities of all hidden states (i.e., $d^{S}$ and $d^{V}$) are set to 512. The scaling thresholds $t_{min}$ and $t_{max}$ are set to 0.5 and 1.0 for Charades-STA and ActivityNet Captions and to 0.3 and 0.7 for TACoS.

For feature encoding, we adopt the pretrained CLIP (ViT-B/32) to jointly encode video and text. All video frames are resized to 224 × 224 prior to encoding. These frames, originally captured by camera-based sensing systems, are treated as visual sensor signals and form the core input to our perception model. The [EOS] token generated by the text encoder serves as the global textual representation, while the visual input for each frame consists of the [CLS] token along with its corresponding patch tokens extracted from the visual encoder.

We use Adam [51] with a learning rate of $1\times 10^{-4}$ and a batch size of 32 for optimization. We adopt the cosine learning rate schedule with a linear warm-up [52] for 5 epochs. In the SVRO module, the Top−K parameter is set to 10. In the TVRO module, both positive and negative frame sequences are sampled with a fixed length of 8. Regarding loss formulation, the cross-modal matching loss and temporal triplet loss are weighted by α=0.3 and β=0.04, respectively.

For the cross-attention module, we adopt a standard multi-head attention mechanism with 8 heads and an attention dimensionality of 512. The query, key, and value projections are implemented as linear layers with identical dimensionality. The subsequent feed-forward network (FFN) consists of a single-layer MLP with a hidden size of 2048, followed by residual connections and layer normalization.

All experiments were conducted using a workstation equipped with an NVIDIA RTX 4090 GPU (NVIDIA Corporation, Santa Clara, CA, USA). The implementation was based on Python 3.8 and PyTorch 1.13.0 (Meta Platforms, Inc., Menlo Park, CA, USA). Video frames were extracted using FFmpeg (Version 4.3.1).

### 4.3. Comparison to State-of-the-Art Methods

#### 4.3.1. Comparative Methods

We compare our proposed method with state-of-the-art video temporal grounding methods on three public datasets. These methods can be grouped into two categories according to the intra-modal and cross-modal viewpoints: (1) The intra-modal methods are 2D-TAN [47], MMN [13], LCNet [14], WSTAN [15], MRTNet [16], D3G [17], CPL [53], CPN [54], DRN [55], GDP [36], and CMIN [56]. (2) The cross-modal methods are LGI [26], PS-VTG [27], VDI [28], CBLN [29], SeqPAN [30], MESM [57], MMDist [58], and VSLNet [35].

#### 4.3.2. Quantitative Comparison

The results are summarized in Table 1, Table 2 and Table 3. Overall, our method outperforms representative state-of-the-art approaches under the most challenging evaluation conditions and consistently ranks among the top three across multiple metrics. On the Charades-STA dataset, it achieves 28.91% on Rank@1, IoU = 0.7 and 42.55% on mIoU, surpassing recent methods such as MMMDist and MRTNet. On ActivityNet Captions, our method obtains the best performance on the Rank@1 metric, particularly achieving 30.36% at IoU = 0.7, underscoring its effectiveness in precise boundary localization. On the TACoS dataset, which is characterized by dense semantic content and significant frame redundancy, our method achieves 31.81% on Rank@1 at IoU = 0.5 and 28.27% on mIoU, ranking second only to CPN and outperforming strong baselines such as SeqPAN and MMN.

To better isolate the source of performance improvements, we conduct a direct comparison with 2D-TAN [47]. Although our method retains the same 2D convolutional architecture for candidate moment modeling and boundary prediction, it consistently outperforms 2D-TAN across all three benchmark datasets. This performance gain cannot be attributed to architectural changes but rather to the enhanced visual representations derived from text-guided optimization during feature construction. By incorporating textual guidance at the encoding stage, our method produces candidate moment representations with improved semantic consistency prior to entering the 2D convolutional network. As a result, the generated 2D temporal feature maps exhibit clearer semantic alignment, leading to overall gains in temporal localization accuracy.

Moreover, we conduct comparative evaluations against several representative intra-modal methods, including MMN, LCNet, WSTAN, MRTNet, and D3G. Our method consistently outperforms these methods across multiple evaluation metrics. This advantage primarily stems from the fact that intra-modal methods do not incorporate semantic priors during visual feature construction. Consequently, their representations tend to lack adaptive sensitivity to textual semantics and rely heavily on late-stage fusion for semantic compensation. Such limitations hinder their ability to effectively handle queries with complex or subtle meanings. In contrast, our method introduces cross-modal semantic guidance at the feature construction stage, enhancing alignment precision and improving temporal localization performance.

Finally, we compare our method with several cross-modal modeling approaches, including CBLN, LGI, PS-VTG, SeqPAN, and VDI. As shown in Table 2, our method achieves superior performance on the ActivityNet Captions dataset. This improvement can be attributed to the inherent complexity of the dataset, which features ambiguous event boundaries, diverse semantic contexts, and significant background clutter. These characteristics pose considerable challenges to a model’s ability to distinguish semantic cues and suppress redundant information. Competing methods rely on frame-level alignment strategies but fail to attend to the most semantically salient subregions within frames. Moreover, they lack dedicated mechanisms for suppressing redundant frames, increasing the likelihood of noise from irrelevant content. In contrast, our method integrates text-guided strategies during feature construction to select semantically aligned patches and emphasize key frames while suppressing irrelevant ones. This leads to more consistent visual representations and improved accuracy in both temporal localization and semantic alignment under complex scenarios.

#### 4.3.3. Plug and Play

We incorporate the proposed visual representation optimization modules into two representative VTG frameworks, MMN [13] and LCNet [14], serving as front-end feature construction components. As reported in Table 4, both models exhibit consistent performance improvements across multiple evaluation metrics on the Charades-STA dataset. These findings demonstrate the modularity and generalizability of our method, confirming its ability to operate as a plug-and-play module that enhances various VTG architectures without requiring architectural redesign. Specifically, MMN constructs a dual-stream semantic matching graph and establishes inter-clip semantic propagation pathways to facilitate the ranking of candidate moments. Its performance is highly dependent on the semantic fidelity of moment-level representations and the discriminative capacity across clips. However, the static visual features utilized by the original MMN often incorporate semantically irrelevant content during candidate moment generation, thereby limiting the representational expressiveness of the constructed graph. By integrating our approach, the SVRO and TVRO modules suppress visual redundancy during the feature construction phase, resulting in candidate moment representations that are more semantically focused. This, in turn, enhances the discriminability of nodes within the semantic graph and improves the effectiveness of the matching process.

As for LCNet, it relies on a weakly supervised cross-frame attention mechanism and a local alignment strategy for clip–text pairs. Through cross-frame semantic aggregation, it learns to approximate the latent temporal boundaries of relevant segments. In this framework, the frame-level semantic granularity of visual features plays a critical role in shaping the attention distribution. However, due to semantic ambiguity and weak activation of salient frames in the original features, the attention mechanism often fails to focus precisely on language-relevant regions. Upon integrating our approach, the TVRO module enhances the alignment between frame-level features and the target query through contrastive supervision. This integration improves the sensitivity of the attention mechanism to text-relevant frames and stabilizes the temporal boundary prediction process.

### 4.4. Ablation Study

#### 4.4.1. Effect of Individual Components

We conduct an ablation study to assess the contribution of each component under the standard experimental setting. Table 5 summarizes the performance variations resulting from the removal of individual modules. The results show that excluding either component leads to a measurable decline in performance, highlighting their respective and complementary roles in spatial and temporal modeling. Specifically, removing the TVRO module causes the mIoU to drop from 44.76% to 41.18%, and it results in a decline of over 4 percentage points in Rank@1 at IoU = 0.7. This underscores the critical role of TVRO in attending to text-relevant frames and suppressing redundant ones. Without it, the model struggles with precise boundary localization and dense semantic alignment. When only SVRO is removed, Rank@1, IoU = 0.3 drops by 3.86%, reflecting a noticeable reduction in the model’s ability to concentrate on salient visual patches within each frame. Its removal directly impairs the spatial grounding of visual representations with respect to the query. When both modules are removed, the model reverts to using unoptimized CLIP features for visual representation construction, resulting in a further mIoU reduction to 39.21%, along with declines in several other metrics. These results indicate that, while CLIP exhibits certain semantic capabilities, its raw visual features still suffer from intra-frame redundancy and limited inter-frame discriminability. By contrast, the combined optimization from SVRO and TVRO produces spatially refined and temporally aligned representations, ultimately yielding superior overall performance.

#### 4.4.2. Effect of Text-Guided Mechanisms in Different Components

To further evaluate the impact of text-guided mechanisms on visual representation construction, we progressively disable three key pathways that incorporate textual supervision. The corresponding results are reported in Table 6. First, removing text guidance in the text-guided patch selection (TGPS) leads to a 3.9% decrease in Rank@1 at IoU = 0.5. This suggests that, without semantic-aligned filtering at the spatial level, redundant visual regions unrelated to the textual query persist, thereby weakening the discriminability of local semantic representations. Additionally, excluding the text-guided aggregator from SVRO results in a further 1.6% performance drop, indicating that patch-level selection alone is insufficient for constructing effective intra-frame semantic focus. The text-guided aggregator plays a critical role in reorganizing spatial semantics and enhancing semantic responsiveness. In the temporal dimension, disabling textual supervision in the TVRO module causes a 4.3% decrease in Rank@1 at IoU = 0.3, reflecting the model’s reduced ability to capture inter-frame semantic density and localize text-relevant key frames. The complete removal of all three text-guided mechanisms causes a substantial drop in overall performance, clearly demonstrating the essential role of text-driven guidance in optimizing visual representations.

#### 4.4.3. Effect of the Number of Salient Patches

As shown in Table 7, we investigate the effect of varying the number of salient patches retained in the SVRO module. The model achieves optimal performance across all evaluation metrics when the Top-K value is set to 10. Under this setting, the selected patches sufficiently capture semantically salient regions while effectively filtering out redundant visual content. When K is further reduced to 5, some key semantic regions are lost, undermining the spatial representational capacity and resulting in a marked decline in model performance. Conversely, increasing K expands semantic coverage but introduces excessive redundancy, which disrupts semantic focus and leads to a consistent decline in precision under stricter IoU thresholds. In the extreme case of retaining all 49 patches without selection, the mIoU drops by 7.6% compared to the optimal setting, indicating that unfiltered input severely harms the model’s discriminative capacity.

#### 4.4.4. Parameter Sensitivity

**Figure 5:**
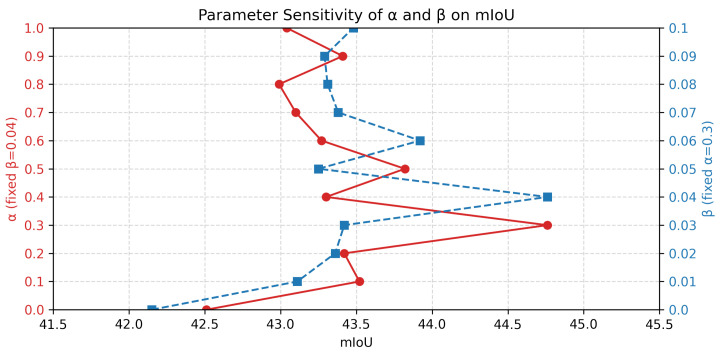
Ablation study of hyperparameter on the ActivityNet Captions dataset. The left Y-axis corresponds to the range of the cross-modal matching loss weight α (with β fixed at 0.04), and the right Y-axis illustrates the range of the temporal triplet loss weight β (with α fixed at 0.3). The horizontal axis reports the corresponding mIoU values.

We conduct two sets of parameter sensitivity experiments to assess the regulatory impact of the cross-modal matching loss weight (α) and the temporal triplet loss weight (β) in Equation (19). Parameter α is varied from 0.0 to 1.0 in increments of 0.1 to examine its influence on the semantic alignment between language queries and clips. Parameter β is tested over the range [0.0, 0.1] with finer granularity to precisely evaluate its effect on temporal modeling through inter-frame contrastive loss. As illustrated by the red curve in Figure 5, setting α=0 eliminates semantic discrimination among clips, leading to a reduction in the mIoU to 42.6%. As α increases to 0.3, the model progressively improves its ability to differentiate between relevant and irrelevant clips, thereby enhancing semantic alignment and raising the mIoU to 44.9%. Further increasing α leads the model to overemphasize semantic similarity at the expense of structural boundary modeling, resulting in the erroneous retention of redundant segments and an increased boundary prediction error, causing a drop in the mIoU. Thus, α=0.3 represents the optimal balance, offering sufficient semantic discrimination without compromising structural fidelity.

The blue curve shows that small values of β fail to enforce sufficient contrast between positive and negative frame sequences, limiting the model’s capacity to identify salient temporal regions, with the mIoU stagnating around 43.1%. Raising β to 0.04 effectively increases the inter-frame representational gap, enabling the model to focus on key semantic frames while suppressing redundant information, thereby improving the mIoU to 44.9%. When β exceeds 0.07, excessive suppression disrupts temporal continuity, causing overly narrow segment localization and boundary misalignment, which ultimately degrades performance.

#### 4.4.5. Effect of Different Visual Features

**Figure 6:**
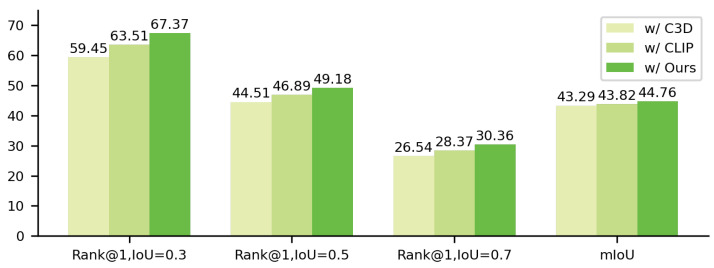
Performance comparison of different visual representations under the 2D-TAN framework.

We conduct comparative experiments on the ActivityNet Captions dataset using three different visual feature configurations within a unified 2D-TAN framework. The results are shown in Figure 6. The first setting employs C3D as the video encoder. Due to the absence of a semantic alignment mechanism, it produces coarse motion features that fail to capture fine-grained correspondences with the query, leading to poor segment localization. The second configuration uses raw features extracted from CLIP. Benefiting from its cross-modal pretraining, it improves overall performance, indicating that semantic embeddings contribute positively to matching quality. However, since CLIP does not filter features based on the specific query, it still suffers from dispersed semantics and redundant visual patches. The third configuration optimizes the CLIP representation with our proposed SVRO and TVRO modules. Without modifying the 2D-TAN architecture, the overall performance is further enhanced. Specifically, Rank@1 at IoU = 0.5 improves by 5.1 percentage points compared to using raw CLIP features. These results underscore the importance of text-guided optimization in enhancing visual representations for the VTG task.

### 4.5. Qualitative Analysis

#### 4.5.1. Inter-Frame Visualization

**Figure 7:**
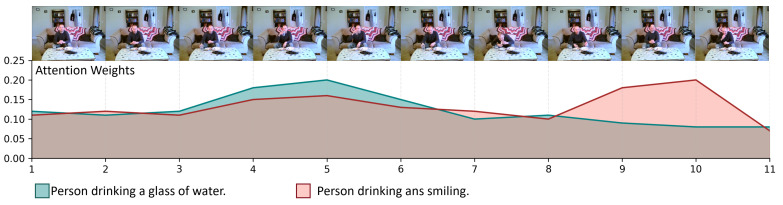
Inter-frame attention comparison across textual queries. We visualize attention weight distributions of 10 frames from the same video clip with respect to two different textual descriptions.

The TVRO module introduces a temporal triplet loss designed to sharpen the model’s focus on frames relevant to the textual query. Figure 7 depicts the frame-wise attention distribution across a single video, evaluated under two different query conditions. The two curves represent queries that are semantically similar yet diverge in their focal intent. The results show that, although both descriptions refer to the same scene, the attention responses vary significantly along the timeline. For the blue query, attention peaks between frames 4 and 6, corresponding closely to the drinking action. In contrast, the pink query elicits heightened attention between frames 9 and 10, highlighting the subject’s facial expression. These results demonstrate the TVRO module’s temporal sensitivity and its ability to adapt the attention distribution based on query semantics, thereby facilitating the precise identification of key semantic frames.

#### 4.5.2. Intra-Frame Visualization

**Figure 8:**
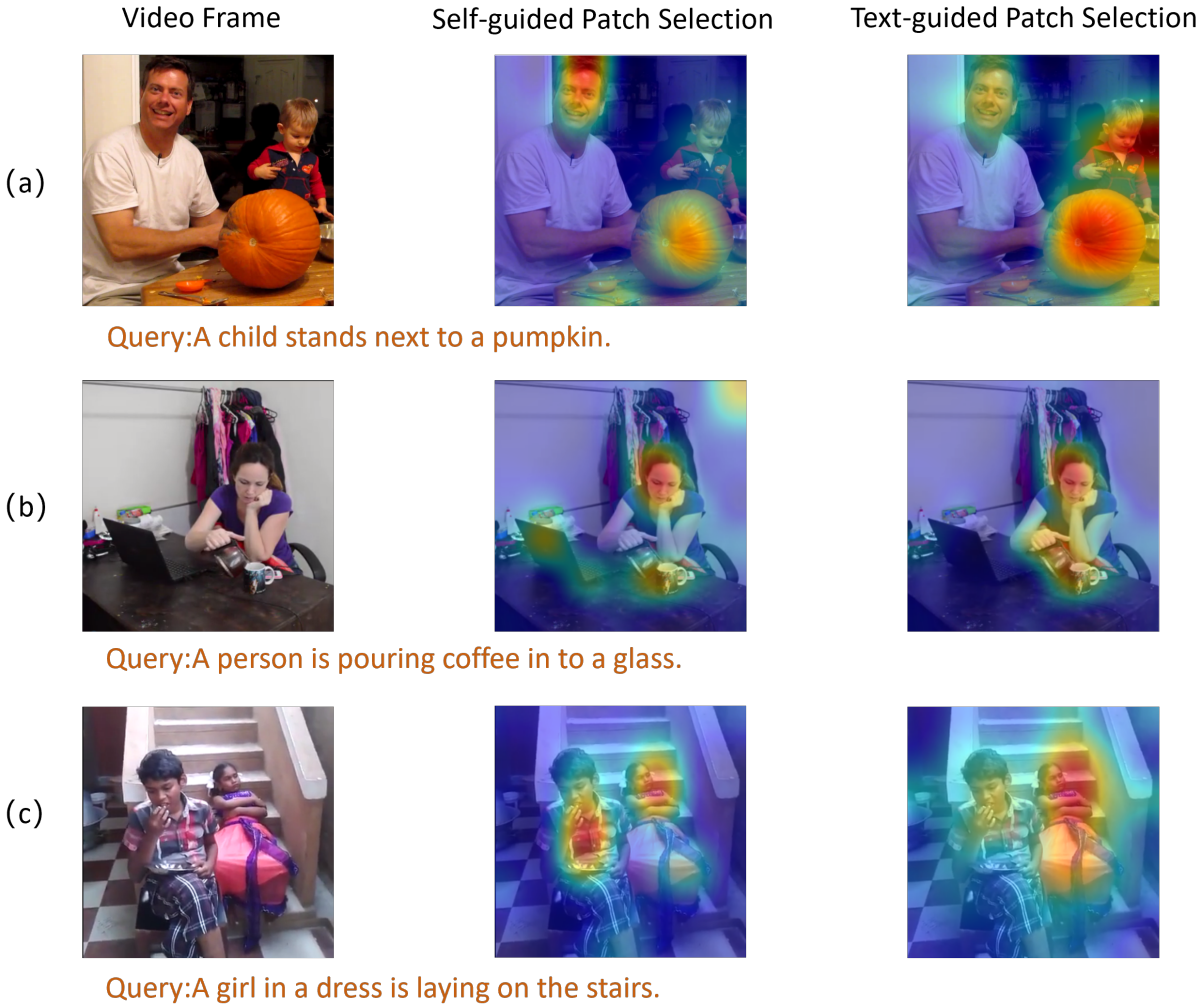
Qualitative comparisons between self-guided and text-guided patch selection.

We conducted a visualization comparing text-guided patch selection with self-guided patch selection derived from image self-attention, as shown in Figure 8. The figure presents three representative vision–language mismatch scenarios, including target omission, weak semantic saliency, and subject ambiguity, which are commonly encountered in real applications. In case (a), the image contains both an adult and a child, and visual self-attention predominantly focuses on facial regions. However, the text description refers only to the “child” and a “pumpkin.” Text-guided patch selection redirects attention from visually dominant regions to semantically aligned areas, effectively filtering out irrelevant content. In case (b), the text highlights the micro-action “pouring coffee,” but the corresponding region is not visually salient. While visual self-attention fails to yield a distinct activation, cross-modal attention amplifies focus on the hand and cup regions, thereby enhancing action localization. In case (c), the scene includes two individuals, but the query refers only to “a girl in a dress.” Visual self-attention distributes attention nearly equally, resulting in reduced discriminability. In contrast, our method suppresses attention to non-target areas via textual cues, ensuring precise alignment with the semantically relevant object.

#### 4.5.3. Grounding Results

**Figure 9:**
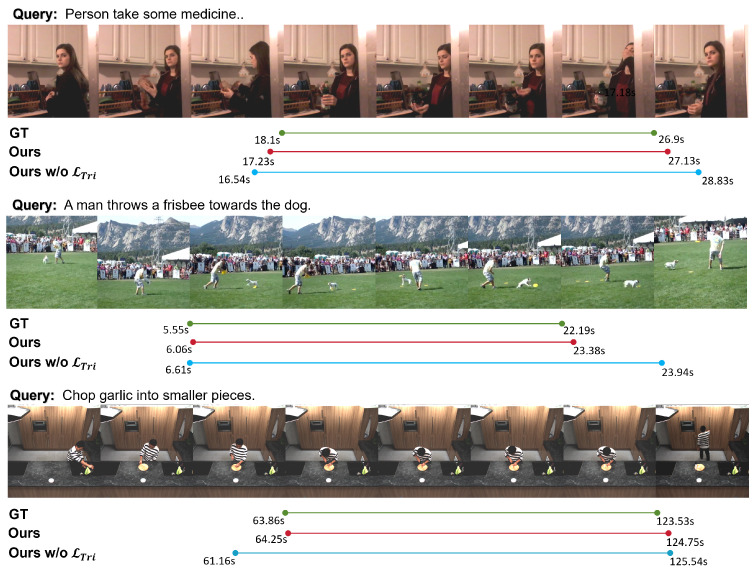
Temporal grounding visualizations on three benchmark datasets.

Figure 9 presents visualizations of the temporal localization results produced by our method on three benchmark datasets. It can be observed that, under various scenes and action semantics, the temporal boundaries predicted by our method align closely with the ground-truth, with boundary positions approximating the actual start and end times of the target described in the text. By contrast, training without the temporal triplet loss causes the model to produce wider temporal spans, with boundaries spilling over into irrelevant frames. This difference indicates that, in the absence of inter-frame semantic contrastive supervision, the model struggles to distinguish between relevant and irrelevant frames in the feature space, resulting in broader, less accurate predictions.

**Figure 10:**
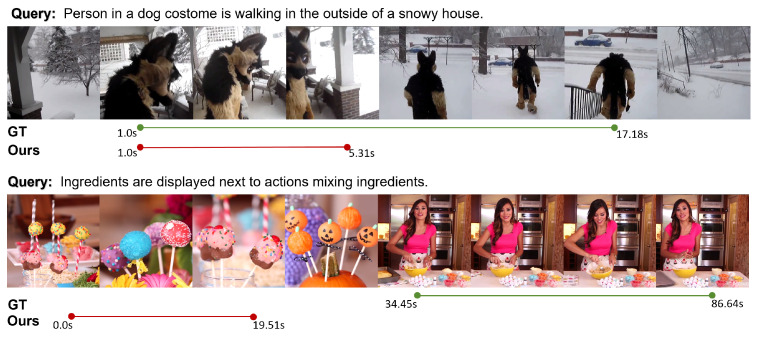
Visualization of failure cases under challenging semantic conditions from ActivityNet Captions.

To further analyze the limitations of our method under challenging conditions, we present two representative failure cases in Figure 10. In the first case, the query describes “a person in a dog costume walking outside a snowy house.” While the sentence conveys a clear action, the textual reference to “a person” conflicts with the animal-like visual appearance, causing a semantic shift across modalities. This mismatch hinders the model’s ability to align the textual and visual representations effectively, leading to incorrect segment selection. In the second case, the query refers to “ingredients displayed next to actions mixing ingredients.” Although this describes a plausible visual scene, the lack of clear temporal cues or visual anchors in the sentence makes it difficult for the model to pinpoint when the actual mixing occurs. As a result, the predicted span fails to capture the intended semantic moment. These cases reveal that, despite the robustness of our method in most scenarios, it still struggles when confronted with semantic ambiguity, cross-modal inconsistency, or underspecified queries. Future work may explore structured semantic decomposition or salient phrase extraction to enhance the model’s grounding capability under complex or underspecified input conditions.

### 4.6. Case Study

#### 4.6.1. Case Study in Challenging Scenarios

**Figure 11:**
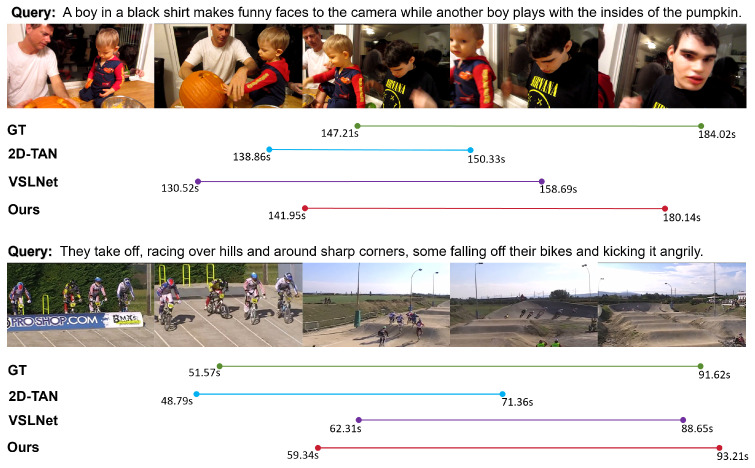
Qualitative comparisons in challenging scenarios from ActivityNet Captions. The first case illustrates the model’s ability to isolate the target activity among concurrent actions, while the second highlights its robustness to rapid scene changes with discontinuous visual content.

In complex multi-activity scenarios, the query often contains multiple semantic units, each referring to a distinct subject and action. This imposes additional demands on the model’s ability to identify and temporally align multiple target behaviors within a densely populated action timeline. As illustrated in the first case of Figure 11, the query describes “a boy in a black shirt making funny faces” and “another boy playing with the insides of the pumpkin,” where the two actions are semantically parallel and temporally overlapping. In such cases, prior methods like 2D-TAN and VSLNet tend to exhibit partial omissions or temporal drift, failing to capture the full scope of the composite semantic expression. By contrast, our method accurately localizes the full extent of the described actions. This improvement is primarily attributed to the semantic alignment capability inherited from CLIP, which offers a unified multimodal embedding space to bridge visual and textual representations. Furthermore, the SVRO module enhances spatial discriminability by filtering out text-irrelevant regions, while the TVRO module suppresses temporally irrelevant frames, allowing the model to retain only the segments that collectively fulfill the query intent. This progressive optimization architecture significantly improves boundary precision in multi-action environments.

In rapidly changing scenes characterized by frequent shot transitions and disrupted action continuity, conventional methods are often prone to spurious responses and imprecise boundaries due to transient distractions. As shown in the second case of Figure 11, our method maintains stable and accurate temporal localization even under such challenging conditions. In comparison, 2D-TAN relies on the direct fusion of visual and textual features without effective temporal disentanglement, limiting its ability to focus on the intended query semantics. VSLNet incorporates fine-grained query guidance, but its uniform frame processing fails to discriminate between informative and redundant content. Our method addresses these challenges through the TVRO module, which dynamically adjusts frame-wise attention weights in accordance with the semantic intensity of the query, thereby reinforcing consistent responses to meaningful segments. Simultaneously, the SVRO module preserves highly correlated regions within individual frames, further sharpening the representational distinctiveness. These mechanisms collectively enable our model to achieve tighter alignment with ground-truth boundaries in rapidly evolving scenes, demonstrating enhanced robustness and generalization capacity.

#### 4.6.2. Case Study Under Environmental Perturbations

**Figure 12:**
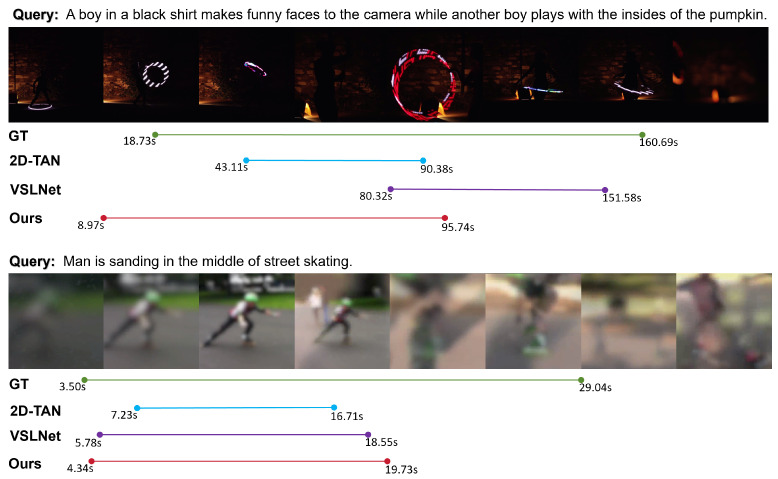
Qualitative comparisons under sensor-induced environmental perturbations from ActivityNet Captions. The first example illustrates a low-light scenario with severe underexposure and missing spatial cues, while the second showcases motion blur caused by rapid object movement.

Environmental degradation in sensor-acquired videos, such as low illumination and motion blur, presents significant challenges to vision–language alignment. In low-light scenarios, underexposed frames often exhibit severe detail loss and diminished contrast, which obscure object boundaries and reduce the availability of semantic cues. As shown in the first case of Figure 12, the scene involves a performer under dim indoor lighting, with the background rendered almost completely dark. This results in a substantial loss of spatial information, making it difficult for visual encoders to capture discriminative features. Both 2D-TAN and VSLNet exhibit marked temporal deviations, either truncating the segment prematurely or overshooting the ground-truth boundaries. While our method does not fully recover the precise extent of the target interval under such adverse sensing conditions, it nonetheless produces significantly closer predictions. This relative robustness stems from the prior alignment knowledge encoded by CLIP during large-scale pretraining, which enhances the model’s sensitivity to semantically relevant frames even when raw sensor input is compromised.

In the second case, motion blur caused by fast object movement leads to widespread contour smearing and a loss of inter-frame consistency. This form of perceptual degradation is common in consumer-grade or mobile sensor setups. As illustrated in Figure 12, the skateboarding sequence suffers from visual ambiguity and reduced spatial clarity. In this setting, 2D-TAN misidentifies the starting point, and VSLNet fails to respond to the final portion of the action. Our method demonstrates more stable temporal predictions by suppressing irrelevant frames and amplifying semantic salience. However, boundary predictions still exhibit slight misalignment, indicating room for improvement in handling ambiguous or low-quality inputs. These cases collectively suggest that, while the proposed framework improves robustness against sensor-induced perturbations compared to prior work, its performance remains sensitive to the severity of visual degradation. Future enhancements may explore adaptive feature compensation strategies or external quality-aware priors to further mitigate sensing limitations.

### 4.7. Computational Complexity Analysis

To gain a more comprehensive understanding of the proposed method, we further analyze its computational complexity by estimating the theoretical floating-point operations (FLOPs) of the two most resource-intensive components: the frame-level cross-modal attention module and the triplet mining mechanism. This analysis not only enables a quantitative assessment of inference load but also provides practical guidance for deployment and resource allocation.

We first examine the computational cost of the frame-level cross-attention mechanism. The module takes as input a sampled visual sequence $v^{*}\in R^{T\times D}$ and a global textual feature $t\in R^{1\times D}$, where *T* is the number of sampled frames, and *D* is the feature dimension. The multi-head attention mechanism consists of *h* parallel subspaces, with each head of dimension $d_{k}=D/h$. According to the standard formulation of attention complexity in Transformer models introduced by Vaswani et al. [59], further elaborated for vision tasks by Dosovitskiy et al. [60], the total FLOPs of the cross-attention operation—including the linear projections for Query, Key, and Value; the attention weight computation with softmax; and the subsequent aggregation—can be approximated by

(20) $${\mathrm{FLOPs}}_{\mathrm{CA}}\approx h\cdot \left(2T\cdot D\cdot d_{k}+2T\cdot d_{k}\right)\approx 2T\cdot D^{2}+O\left(T\cdot D\right)$$

Under the experimental setup of D=512, h=8, and thus $d_{k}=64$, with sampled sequences comprising 8 frames from each of the positive, negative, and mixed types, we have T=24. Substituting into the equation yields approximately $1.26\times 10^{7}$ FLOPs. The following feed-forward network (FFN), consisting of two linear transformations with a hidden dimension of 2048, incurs an additional cost of

(21) $${\mathrm{FLOPs}}_{\mathrm{FFN}}\approx 2T\cdot D\cdot 2048\approx 5.03\times 10^{7}$$

Thus, the total cost for a single invocation of the frame-level cross-attention, including FFN, amounts to approximately 0.063 GFLOPs. It is worth noting that this module operates only over a fixed set of sampled frames rather than the full video, ensuring that the overall computational burden remains manageable. We now turn to the triplet mining module, which aims to enhance the discriminability of temporal feature alignment. Each training instance generates $n_{tri}$ triplets based on the sampled positive and negative frame sequences, each comprising a hybrid frame feature $v_{t}^{hyb}$, a positive sample $v_{t}^{pos}$, and a negative sample $v_{t}^{neg}$. Following the design of FaceNet by Schroff et al. [61], each triplet involves two Euclidean distance computations and a subsequent normalization operation, with the overall complexity approximately given by

(22) $${\mathrm{FLOPs}}_{\mathrm{Tri}}\approx n_{\mathrm{tri}}\cdot \left(c_{1}\cdot D+c_{2}\right)=O\left(n_{\mathrm{tri}}\cdot D\right)$$

For $n_{tri}=50$ and D=512, the total cost is below $10^{6}$ FLOPs (less than 0.001 GFLOPs) and, thus, can be regarded as a negligible overhead.

In summary, the combined computational cost of the text-aggregator and temporal triplet loss modules remains under 0.064 GFLOPs, significantly lower than that of visual encoders, proposal generators, or common multi-scale attention structures. More importantly, the proposed framework adopts a sparsely sampled frame interaction strategy with local attention design, which scales linearly with the video length. This design effectively avoids the quadratic or cubic complexity explosion typically observed in dense temporal modeling. As a result, even when applied to long-duration videos or batch-level inference, the system maintains controllable inference efficiency and memory usage by adjusting the sampling interval and interaction granularity.

## 5. Conclusions

This study investigates the challenge of inadequate cross-modal alignment and deficient semantic responsiveness in visual representations for video temporal grounding and proposes a text-guided visual representation optimization framework to address these limitations. The framework builds upon CLIP to construct a unified visual–text embedding space and incorporates two modules, SVRO and TVRO, to identify spatially and temporally salient content that aligns with the semantic intent of the query. These visual signals originate from camera-based sensing systems, and our method enhances their interpretability through multimodal semantic conditioning.

In particular, the SVRO module performs fine-grained patch-level filtering to extract text-relevant spatial regions, while the TVRO module dynamically suppresses temporally irrelevant frames, ensuring precise alignment with the query’s semantic focus. This dual-stage optimization significantly improves the expressiveness and discriminability of visual features across challenging scenarios, including multi-activity compositions, rapid scene transitions, and sensor-induced degradation. The incorporation of cross-modal contrastive loss further boosts the discriminative capability of the segment-level representations, enhancing query-aware localization accuracy. Extensive experiments on three public benchmarks demonstrate consistent improvements over representative methods, validating the effectiveness of the proposed framework. Furthermore, its integration into diverse VTG architectures confirms its adaptability and transferability. This work contributes to intelligent visual sensing by optimizing the semantic understanding of sensor-acquired video content through a novel combination of pre-trained multimodal encoders and task-specific semantic filtering strategies.

Future work may explore structured semantic decomposition or salient phrase extraction to enhance the model’s grounding capability under complex or underspecified input conditions. Moreover, continued progress may focus on adaptive feature compensation strategies or external quality-aware priors to improve robustness under visual degradation, thereby broadening applicability in real-world scenarios involving ambiguous, incomplete, or low-quality sensor inputs.

## Figures and Tables

**Table 1:** Performance comparison with the state-of-the-art models on the Charades-STA dataset. The upward arrow (↑) indicates that higher values are better.

Methods	Publication	Rank@1			mIoU ↑
		IoU = 0.3 ↑	IoU = 0.5 ↑	IoU = 0.7 ↑	
2D-TAN [47]	AAAI2020	57.31	39.70	23.31	39.23
DRN [55]	CVPR2020	—	42.90	23.68	—
GDP [36]	AAAI2020	54.54	39.47	18.49	—
CBLN [29]	CVPR2021	—	43.67	24.44	—
CPN [54]	CVPR2021	64.41	46.08	25.06	43.90
LCNet [14]	ITIP2021	59.60	39.19	18.87	38.94
WSTAN [15]	ITM2022	43.39	29.35	12.28	—
CPL [53]	CVPR2022	66.40	49.24	22.39	—
MMN [13]	AAAI2022	—	47.41	27.28	—
PS-VTG [27]	ITM2022	60.40	39.22	20.17	39.77
D3G [17]	ICCV2023	—	41.64	19.60	—
VDI [28]	CVPR2023	—	46.47	28.63	41.60
MESM [57]	AAAI2024	—	56.69	35.99	37.33
MMDist [58]	AAAI2024	67.26	51.58	24.22	—
MRTNet [16]	ICASSP2024	59.23	44.27	25.88	40.59
Ours	—	65.54	45.83	28.91	42.55

**Table 2:** Performance comparison with the state-of-the art models on the ActivityNet Captions dataset.

Methods	Publication	Rank@1			mIoU ↑
		IoU = 0.3 ↑	IoU = 0.5 ↑	IoU = 0.7 ↑	
2D-TAN [47]	AAAI2020	59.45	44.51	26.54	43.29
DRN [55]	CVPR2020	58.52	41.51	23.07	43.13
GDP [36]	AAAI2020	56.17	39.27	—	39.80
LGI [26]	CVPR2020	58.52	41.51	23.07	41.13
CBLN [29]	CVPR2021	66.34	48.12	27.60	—
LCNet [14]	ITIP2021	48.49	26.33	—	34.29
CPN [54]	CVPR2021	62.81	45.10	28.10	—
SeqPAN [30]	ACL2021	61.65	45.50	28.37	45.11
WSTAN [15]	ITM2022	52.45	30.01	—	—
CPL [53]	CVPR2022	55.73	31.14	—	—
MMN [13]	AAAI2022	65.05	48.59	29.26	—
PS-VTG [27]	ITM2022	59.71	39.59	21.98	41.49
D3G [17]	ICCV2023	58.25	36.68	18.54	—
VDI [28]	CVPR2023	—	32.35	16.02	34.32
MMDist [58]	AAAI2024	56.92	31.80	—	—
MRTNet [16]	ICASSP2024	60.71	45.59	28.07	44.54
Ours	—	66.37	49.18	30.36	45.76

**Table 3:** Performance comparison with the state-of-the-art models on the TACoS dataset.

Methods	Publication	Rank@1			mIoU ↑
		IoU = 0.3 ↑	IoU = 0.5 ↑	IoU = 0.7 ↑	
2D-TAN [47]	AAAI2020	37.29	25.32	13.32	25.19
VSLNet [35]	ACL2020	29.61	24.27	20.03	24.11
CMIN [56]	SIGIR2020	32.35	22.54	—	—
CBLN [29]	CVPR2021	38.98	27.65	—	—
CPN [54]	CVPR2021	47.69	36.33	21.58	34.49
SeqPAN [30]	ACL2021	31.72	27.19	21.65	25.86
MMN [13]	AAAI2022	38.57	27.24	—	—
PS-VTG [27]	ITM2022	23.64	10.00	3.35	17.39
D3G [17]	ICCV2023	27.27	12.67	4.70	—
MRTNet [16]	ICASSP2024	37.81	26.01	14.95	26.29
Ours	—	45.33	31.81	18.15	28.27

**Table 4:** We serve our method as a plug-and-play module for state-of-the-art VTG methods on Charades-STA under official train/test splits.

Methods	Variant	Rank@1			mIoU ↑
		IoU = 0.3 ↑	IoU = 0.5 ↑	IoU = 0.7 ↑	
MMN	Origin	—	47.31	29.28	—
	+Ours	66.03	49.14	30.21	44.20
LCNet	Origin	59.60	39.19	18.87	38.94
	+Ours	62.45	41.52	20.12	39.88

**Table 5:** Ablation study of individual components on the ActivityNet Captions dataset. ✓ and ✗ denote the presence and absence of the corresponding component.

Components		Rank@1			mIoU ↑
SRVO	TVRO	IoU = 0.3 ↑	IoU = 0.5 ↑	IoU = 0.7 ↑	
✗	✓	63.51	46.89	28.37	42.32
✓	✗	64.78	45.17	26.02	41.18
✗	✗	61.42	43.33	25.51	39.21
✓	✓	66.37	49.18	30.36	45.76

**Table 6:** Ablation results of text-guided mechanisms in different components on the ActivityNet Captions dataset. TGPS refers to the text-guided patch selection strategy employed during spatial representation learning. ✓ indicates that the text-guided mechanism is enabled for the corresponding module; ✗ indicates that it is disabled.

TGPS	SRVO	TVRO	Rank@1			mIoU ↑
Text Guidance			IoU = 0.3 ↑	IoU = 0.5 ↑	IoU = 0.7 ↑	
✗	✗	✓	63.02	45.26	27.38	41.16
✓	✓	✗	64.31	45.87	27.01	41.28
✓	✗	✓	63.29	44.28	26.10	40.55
✓	✓	✓	67.37	49.18	30.36	44.76

**Table 7:** Ablation study of the number of salient patches on the ActivityNet Captions dataset. The highest metric is emphasized in bold. CLIP (ViT-B/32) is utilized as the backbone. The 224×224 image is divided into 49 patches.

Salient Patches	Rank@1			mIoU ↑
	IoU = 0.3 ↑	IoU = 0.5 ↑	IoU = 0.7 ↑	
K=5	66.12	48.36	29.59	44.02
K=10	**67.37**	**49.18**	**30.36**	**44.76**
K=15	66.21	48.20	29.74	44.05
K=20	66.28	47.83	29.11	44.12
K=49 (all)	62.93	45.10	26.97	41.35

## Data Availability

The data used in this study are publicly available from the corresponding benchmark datasets: Charades-STA, ActivityNet Captions, and TACoS. Processed data and implementation details supporting the findings of this work are available from the corresponding author upon reasonable request.

## References

[1] Wang L., Li W., Li W., Van Gool L. Appearance-and-Relation Networks for Video Classification. Proceedings of the 2018 IEEE/CVF Conference on Computer Vision and Pattern Recognition.

[2] Feichtenhofer C., Fan H., Malik J., He K. SlowFast Networks for Video Recognition. Proceedings of the 2019 IEEE/CVF International Conference on Computer Vision (ICCV).

[3] Zhao Y., Xiong Y., Wang L., Wu Z., Tang X., Lin D. Temporal Action Detection with Structured Segment Networks. Proceedings of the 2017 IEEE International Conference on Computer Vision (ICCV).

[4] Lin Z., Zhao Z., Zhang Z., Zhang Z., Cai D. (2020). Moment Retrieval via Cross-Modal Interaction Networks with Query Reconstruction. IEEE Trans. Image Process..

[5] Gao J., Sun C., Yang Z., Nevatia R. TALL: Temporal Activity Localization via Language Query. Proceedings of the 2017 IEEE International Conference on Computer Vision (ICCV).

[6] Chu Y.W., Lin K.Y., Hsu C.C., Ku L.W. (2021). End-to-End Recurrent Cross-Modality Attention for Video Dialogue. IEEE/ACM Trans. Audio, Speech, Lang. Process..

[7] Ji W., Li Y., Wei M., Shang X., Xiao J., Ren T., Chua T.S. VidVRD 2021: The Third Grand Challenge on Video Relation Detection. Proceedings of the 29th ACM International Conference on Multimedia, MM’21.

[8] Shang X., Li Y., Xiao J., Ji W., Chua T.S. Video Visual Relation Detection via Iterative Inference. Proceedings of the 29th ACM International Conference on Multimedia, MM’21.

[9] Shang X., Ren T., Guo J., Zhang H., Chua T.S. Video Visual Relation Detection. Proceedings of the 25th ACM International Conference on Multimedia, MM’17.

[10] Li Y., Wang X., Xiao J., Ji W., Chua T.S. Invariant Grounding for Video Question Answering. Proceedings of the 2022 IEEE/CVF Conference on Computer Vision and Pattern Recognition (CVPR).

[11] Xiao J., Yao A., Liu Z., Li Y., Ji W., Chua T.S. (2022). Video as Conditional Graph Hierarchy for Multi-Granular Question Answering. Proc. AAAI Conf. Artif. Intell..

[12] Zhong Y., Ji W., Xiao J., Li Y., Deng W., Chua T.S. Video Question Answering: Datasets, Algorithms and Challenges. Proceedings of the 2022 Conference on Empirical Methods in Natural Language Processing.

[13] Wang Z., Wang L., Wu T., Li T., Wu G. (2021). Negative Sample Matters: A Renaissance of Metric Learning for Temporal Grounding. arXiv.

[14] Yang W., Zhang T., Zhang Y., Wu F. (2021). Local Correspondence Network for Weakly Supervised Temporal Sentence Grounding. IEEE Trans. Image Process..

[15] Wang Y., Deng J., Zhou W., Li H. (2022). Weakly Supervised Temporal Adjacent Network for Language Grounding. IEEE Trans. Multimed..

[16] Ji W., Qin Y., Chen L., Wei Y., Wu Y., Zimmermann R. Mrtnet: Multi-Resolution Temporal Network for Video Sentence Grounding. Proceedings of the ICASSP 2024—2024 IEEE International Conference on Acoustics, Speech and Signal Processing (ICASSP).

[17] Li H., Shu X., He S., Qiao R., Wen W., Guo T., Gan B., Sun X. D3G: Exploring Gaussian Prior for Temporal Sentence Grounding with Glance Annotation. Proceedings of the 2023 IEEE/CVF International Conference on Computer Vision (ICCV).

[18] Gorti S.K., Vouitsis N., Ma J., Golestan K., Volkovs M., Garg A., Yu G. X-Pool: Cross-Modal Language-Video Attention for Text-Video Retrieval. Proceedings of the 2022 IEEE/CVF Conference on Computer Vision and Pattern Recognition (CVPR).

[19] Guan P., Pei R., Shao B., Liu J., Li W., Gu J., Xu H., Xu S., Yan Y., Lam E.Y. PIDRo: Parallel Isomeric Attention with Dynamic Routing for Text-Video Retrieval. Proceedings of the 2023 IEEE/CVF International Conference on Computer Vision (ICCV).

[20] Liu Y., Xiong P., Xu L., Cao S., Jin Q. (2022). TS2-Net: Token Shift and Selection Transformer for Text-Video Retrieval. Computer Vision—ECCV 2022, Proceedings of the 17th European Conference, Tel Aviv, Israel, 23–27 October 2022.

[21] Wang Z., Sung Y.L., Cheng F., Bertasius G., Bansal M. Unified Coarse-to-Fine Alignment for Video-Text Retrieval. Proceedings of the 2023 IEEE/CVF International Conference on Computer Vision (ICCV).

[22] Krishna R., Hata K., Ren F., Fei-Fei L., Niebles J.C. Dense-Captioning Events in Videos. Proceedings of the 2017 IEEE International Conference on Computer Vision (ICCV).

[23] Rohrbach M., Regneri M., Andriluka M., Amin S., Pinkal M., Schiele B. Script data for attribute-based recognition of composite activities. Proceedings of the 12th European Conference on Computer Vision ECCV’12.

[24] Lin T., Zhao X., Shou Z. Single Shot Temporal Action Detection. Proceedings of the 25th ACM International Conference on Multimedia, MM’17.

[25] Hendricks L.A., Wang O., Shechtman E., Sivic J., Darrell T., Russell B. Localizing Moments in Video with Natural Language. Proceedings of the 2017 IEEE International Conference on Computer Vision (ICCV).

[26] Mun J., Cho M., Han B. Local-Global Video-Text Interactions for Temporal Grounding. Proceedings of the 2020 IEEE/CVF Conference on Computer Vision and Pattern Recognition (CVPR).

[27] Xu Z., Wei K., Yang X., Deng C. (2023). Point-Supervised Video Temporal Grounding. IEEE Trans. Multimed..

[28] Luo D., Huang J., Gong S., Jin H., Liu Y. Towards Generalisable Video Moment Retrieval: Visual-Dynamic Injection to Image-Text Pre-Training. Proceedings of the 2023 IEEE/CVF Conference on Computer Vision and Pattern Recognition (CVPR).

[29] Liu D., Qu X., Dong J., Zhou P., Cheng Y., Wei W., Xu Z., Xie Y. Context-aware Biaffine Localizing Network for Temporal Sentence Grounding. Proceedings of the 2021 IEEE/CVF Conference on Computer Vision and Pattern Recognition (CVPR).

[30] Zhang H., Sun A., Jing W., Zhen L., Zhou J.T., Goh S.M.R. Parallel Attention Network with Sequence Matching for Video Grounding. Proceedings of the Findings of the Association for Computational Linguistics: ACL-IJCNLP 2021, Association for Computational Linguistics.

[31] Chen Y.W., Tsai Y.H., Wang T., Lin Y.Y., Yang M.H. (2019). Referring Expression Object Segmentation with Caption-Aware Consistency. arXiv.

[32] Chen Y.W., Tsai Y.H., Yang M.H. (2021). Understanding Synonymous Referring Expressions via Contrastive Features. arXiv.

[33] Kim J., Ma M., Pham T., Kim K., Yoo C.D. Modality Shifting Attention Network for Multi-Modal Video Question Answering. Proceedings of the 2020 IEEE/CVF Conference on Computer Vision and Pattern Recognition (CVPR).

[34] Gheini M., Ren X., May J. (2021). Cross-Attention is All You Need: Adapting Pretrained Transformers for Machine Translation. arXiv.

[35] Zhang H., Sun A., Jing W., Zhou J.T. (2020). Span-based Localizing Network for Natural Language Video Localization. arXiv.

[36] Chen L., Lu C., Tang S., Xiao J., Zhang D., Tan C., Li X. (2020). Rethinking the Bottom-Up Framework for Query-Based Video Localization. Proc. AAAI Conf. Artif. Intell..

[37] Wang J., Gong T., Zeng Z., Sun C., Yan Y. C3CMR: Cross-Modality Cross-Instance Contrastive Learning for Cross-Media Retrieval. Proceedings of the 30th ACM International Conference on Multimedia, MM’22.

[38] Ma Y., Xu G., Sun X., Yan M., Zhang J., Ji R. X-CLIP: End-to-End Multi-grained Contrastive Learning for Video-Text Retrieval. Proceedings of the 30th ACM International Conference on Multimedia, MM’22.

[39] Yuan L., Qian R., Cui Y., Gong B., Schroff F., Yang M.H., Adam H., Liu T. Contextualized Spatio-Temporal Contrastive Learning with Self-Supervision. Proceedings of the 2022 IEEE/CVF Conference on Computer Vision and Pattern Recognition (CVPR).

[40] Ding S., Qian R., Xiong H. Dual Contrastive Learning for Spatio-temporal Representation. Proceedings of the 30th ACM International Conference on Multimedia, MM ’22.

[41] Radford A., Kim J.W., Hallacy C., Ramesh A., Goh G., Agarwal S., Sastry G., Askell A., Mishkin P., Clark J. Learning Transferable Visual Models From Natural Language Supervision. Proceedings of the 38th International Conference on Machine Learning.

[42] Luo H., Ji L., Zhong M., Chen Y., Lei W., Duan N., Li T. (2021). CLIP4Clip: An Empirical Study of CLIP for End to End Video Clip Retrieval. arXiv.

[43] Xue H., Sun Y., Liu B., Fu J., Song R., Li H., Luo J. (2023). CLIP-ViP: Adapting Pre-trained Image-Text Model to Video-Language Representation Alignment. arXiv.

[44] Tian Y., Guo X., Wang J., Li B. (2025). Enhancing video temporal grounding with large language model-based data augmentation. J. Supercomput..

[45] Ibrahimi S., Sun X., Wang P., Garg A., Sanan A., Omar M. Audio-Enhanced Text-to-Video Retrieval using Text-Conditioned Feature Alignment. Proceedings of the 2023 IEEE/CVF International Conference on Computer Vision (ICCV).

[46] Jin P., Li H., Cheng Z., Huang J., Wang Z., Yuan L., Liu C., Chen J. Text-video retrieval with disentangled conceptualization and set-to-set alignment. Proceedings of the Thirty-Second International Joint Conference on Artificial Intelligence, IJCAI ’23.

[47] Zhang S., Peng H., Fu J., Luo J. (2020). Learning 2D Temporal Adjacent Networks for Moment Localization with Natural Language. arXiv.

[48] Sigurdsson G.A., Varol G., Wang X., Farhadi A., Laptev I., Gupta A. (2016). Hollywood in Homes: Crowdsourcing Data Collection for Activity Understanding. arXiv.

[49] Heilbron F.C., Escorcia V., Ghanem B., Niebles J.C. ActivityNet: A large-scale video benchmark for human activity understanding. Proceedings of the 2015 IEEE Conference on Computer Vision and Pattern Recognition (CVPR).

[50] Regneri M., Rohrbach M., Wetzel D., Thater S., Schiele B., Pinkal M. (2013). Grounding Action Descriptions in Videos. Trans. Assoc. Comput. Linguist..

[51] Kingma D.P. (2014). Adam: A method for stochastic optimization. arXiv.

[52] Goyal P., Dollár P., Girshick R., Noordhuis P., Wesolowski L., Kyrola A., Tulloch A., Jia Y., He K. (2018). Accurate, Large Minibatch SGD: Training ImageNet in 1 Hour. arXiv.

[53] Zheng M., Huang Y., Chen Q., Peng Y., Liu Y. Weakly Supervised Temporal Sentence Grounding with Gaussian-based Contrastive Proposal Learning. Proceedings of the 2022 IEEE/CVF Conference on Computer Vision and Pattern Recognition (CVPR).

[54] Zhao Y., Zhao Z., Zhang Z., Lin Z. Cascaded Prediction Network via Segment Tree for Temporal Video Grounding. Proceedings of the 2021 IEEE/CVF Conference on Computer Vision and Pattern Recognition (CVPR).

[55] Zeng R., Xu H., Huang W., Chen P., Tan M., Gan C. Dense Regression Network for Video Grounding. Proceedings of the 2020 IEEE/CVF Conference on Computer Vision and Pattern Recognition (CVPR).

[56] Zhang Z., Lin Z., Zhao Z., Xiao Z. Cross-Modal Interaction Networks for Query-Based Moment Retrieval in Videos. Proceedings of the 42nd International ACM SIGIR Conference on Research and Development in Information Retrieval, SIGIR’19.

[57] Liu Z., Li J., Xie H., Li P., Ge J., Liu S.A., Jin G. Towards balanced alignment: Modal-enhanced semantic modeling for video moment retrieval. Proceedings of the Thirty-Eighth AAAI Conference on Artificial Intelligence and Thirty-Sixth Conference on Innovative Applications of Artificial Intelligence and Fourteenth Symposium on Educational Advances in Artificial Intelligence, AAAI’24/IAAI’24/EAAI’24.

[58] Bao P., Xia Y., Yang W., Ng B.P., Er M.H., Kot A.C. Local-global multi-modal distillation for weakly-supervised temporal video grounding. Proceedings of the Thirty-Eighth AAAI Conference on Artificial Intelligence and Thirty-Sixth Conference on Innovative Applications of Artificial Intelligence and Fourteenth Symposium on Educational Advances in Artificial Intelligence, AAAI’24/IAAI’24/EAAI’24.

[59] Vaswani A., Shazeer N., Parmar N., Uszkoreit J., Jones L., Gomez A.N., Kaiser L.u., Polosukhin I. Attention is All you Need. Proceedings of the Advances in Neural Information Processing Systems.

[60] Dosovitskiy A., Beyer L., Kolesnikov A., Weissenborn D., Zhai X., Unterthiner T., Dehghani M., Minderer M., Heigold G., Gelly S. (2021). An Image is Worth 16 × 16 Words: Transformers for Image Recognition at Scale. arXiv.

[61] Schroff F., Kalenichenko D., Philbin J. FaceNet: A unified embedding for face recognition and clustering. Proceedings of the 2015 IEEE Conference on Computer Vision and Pattern Recognition (CVPR).

